# Evaluating Diagnostic Concordance in Primary Open-Angle Glaucoma Among Academic Glaucoma Subspecialists

**DOI:** 10.3390/diagnostics14212460

**Published:** 2024-11-03

**Authors:** Chenmin Wang, De-Fu Chen, Xiao Shang, Xiaoyan Wang, Xizhong Chu, Chengju Hu, Qiangjie Huang, Gangwei Cheng, Jianjun Li, Ruiyi Ren, Yuanbo Liang

**Affiliations:** 1 National Clinical Research Center for Ocular Diseases, Eye Hospital, Wenzhou Medical University, Wenzhou 325000, China; cboy2014@foxmail.com (C.W.); ysgcdf@wmu.edu.cn (D.-F.C.); lulu_xiaoshang@outlook.com (X.S.); xiaoyan_eye@126.com (X.W.); wmu_chuxizhong1997@163.com (X.C.); chengju_hu@outlook.com (C.H.); hqj12@eye.ac.cn (Q.H.); renruiyi@eye.ac.cn (R.R.); 2 Peking Union Medical College Hospital, Beijing 100730, China; chenggw@pumch.cn; 3 Beijing Tongren Eye Center, Beijing Tongren Hospital, Capital Medical University, Beijing 100730, China; jianjunli2005@163.com

**Keywords:** glaucoma, diagnosis, agreement

## Abstract

**Objective:** The study aimed to evaluate the interobserver agreement among glaucoma subspecialists in diagnosing glaucoma and to explore the causes of diagnostic discrepancies. **Methods:** Three experienced glaucoma subspecialists independently assessed frequency domain optical coherence tomography, fundus color photographs, and static perimetry results from 464 eyes of 275 participants, adhering to unified glaucoma diagnostic criteria. All data were collected from the Wenzhou Glaucoma Progression Study between August 2014 and June 2021. **Results:** The overall interobserver agreement among the three experts was poor, with a Fleiss’ kappa value of 0.149. The kappa values interobserver agreement between pairs of experts ranged from 0.133 to 0.282. In 50 cases, or approximately 10.8%, the three experts reached completely different diagnoses. Agreement was more likely in cases involving larger average cup-to-disc ratios, greater vertical cup-to-disc ratios, more severe visual field defects, and thicker retinal nerve fiber layer measurements, particularly in the temporal and inferior quadrants. High myopia also negatively impacted interobserver agreement. **Conclusions:** Despite using unified diagnostic criteria for glaucoma, significant differences in interobserver consistency persist among glaucoma subspecialists. To improve interobserver agreement, it is recommended to provide additional training on standardized diagnostic criteria. Furthermore, for cases with inconsistent diagnoses, long-term follow-up is essential to confirm the diagnosis of glaucoma.

## 1. Introduction

Glaucoma, a condition marked by progressive optic neuropathy, is the leading cause of irreversible blindness worldwide [1]. It affected approximately 76 million people aged 40 to 80 years in 2020, with this number expected to rise to 111.8 million by 2040 [2,3]. Although lowering intraocular pressure (IOP) can prevent or slow the progression of glaucoma [4,5], the rates of blindness due to glaucoma remain high, ranging from 13.5% to 26.5% over a 10-year period [4,6,7]. The risk of developing blindness increases with the severity of visual field (VF) defects at the time of diagnosis [8]. Therefore, early detection and treatment are essential for preserving residual visual function and improving patients’ quality of life [9,10].

The diagnosis of glaucoma typically involves a combination of functional and structural assessments. The VF test, commonly performed using static automated perimetry, evaluates functional impairment, while optic disc fundus stereo photographs have traditionally been used to assess structural damage [11,12]. Additionally, optical coherence tomography (OCT) is increasingly used to confirm structural damage and detect early optic neuropathy [13,14,15].

One of the major challenges in diagnosing glaucoma is the absence of a universally accepted criteria for evaluating glaucomatous optic neuropathy. Even when following the same guidelines, differences in clinicians’ experience and subjective interpretations often lead to inconsistent diagnoses, particularly in the early stages of the disease. Previous studies have reported moderate interobserver agreement in glaucoma diagnosis, with kappa values ranging from 0.40~0.68 [16,17,18,19]. In a study by Hood et al. [17], the diagnoses made by three glaucoma experts were inconsistent in 40% of cases, even when disc photographs, VF, and OCT data were available. Similarly, Blumberg et al. [18] found poor interobserver agreement in detecting glaucomatous damage when using disc photographs, VF, and OCT independently. Among these, the highest interobserver agreement was observed with OCT interpretations (kappa = 0.40), while the agreements for interpreting VF and fundus photography were lower, with kappa values of 0.13 and 0.16, respectively.

Most previous studies have primarily analyzed interobserver agreement in detecting glaucomatous changes using only a single modality, such as optic disc changes on fundus photographs, VF results, or OCT findings. However, in clinical practice, all of these examinations are considered collectively when diagnosing glaucoma. Additionally, studies that included fundus photographs, VF, and OCT results often had relatively small sample sizes.

Therefore, this study aimed to compare the interobserver agreement among glaucoma subspecialists using a combination of frequency domain OCT, fundus photographs, and perimetry results. The goal was to better understand the variability in glaucoma diagnosis and provide guidance for clinical practice.

## 2. Materials and Methods

### 2.1. Study Design

The Wenzhou Glaucoma Progression Study (WPGS) is a prospective longitudinal cohort study conducted at the Eye Hospital of Wenzhou Medical University, offering free glaucoma screenings to the Wenzhou community. Participants were recruited from WPGS between August 2014 and June 2021, with initial screenings conducted by two residents from the Eye Hospital of Wenzhou Medical University. Individuals with suspected glaucoma were included in the study, except those who met any of the following exclusion criteria: (1) acute angle-closure glaucoma; (2) inability to complete the ophthalmic examination; or (3) poor image quality of fundus photographs or unreliable VF results.

Written informed consent was obtained from all participants. The study was approved by the Ethics Committee of the Wenzhou Medical University (KYK 2013] No. 12) and adhered to the principles outlined in the Declaration of Helsinki.

### 2.2. Methods

Demographic data and medical history were collected for all enrolled participants, along with a variety of ophthalmic examinations. These included measurements of IOP, fundus photography, OCT, VF tests, gonioscopy, and axial length (AL), among others.

IOP was measured using Goldmann applanation tonometry (HAAG-STREIT 900 CM; HaagStreit, Kloniz, Switzerland). IOP was measured twice, and if the two measurements differed by more than 2 mmHg, a third measurement was taken. The mean of the two or three IOP measurements was used in the analyses.

Fundus color photographs were taken with a VISUCAM 200 (Zeiss, Oberkochen, Germany), focusing on the optic disc. The examination was conducted in a dark environment, with pupil dilation using MIDORI or tropicamide phenylephrine eye drops if necessary. High-quality optic disc photographs were selected based on the following criteria: (1) the image center was positioned between the optic disc and the macula; (2) the images included the optic disc, macular area, and retinal vascular arches for comprehensive assessment; and (3) the photographs were properly focused and had appropriate exposure to clearly distinguish small blood vessels and the retinal nerve fiber layer on the optic disc.

VF examinations were conducted using the Humphrey fully automatic visual field analyzer (Carl Zeiss 750 or 740, Zeiss, Germany) with the SITA-Standard mode (24-2). Only reliable VF results were included, defined by a fixation loss rate of less than 20% and a false positive rate of less than 15%. The first test was excluded to minimize the impact of the learning effect.

OCT examinations were performed using the Cirrus HD-OCT (ivue, Zeiss, Germany). Images with a scan quality below 6/10 or of poor quality were excluded. Optic disc morphological parameters were analyzed using computerized imaging analysis. Peripapillary RNFL thickness were calculated using the 3.46 mm circle scan diameter centered on the optic disc. Global and quadrant peripapillary RNFL thickness were automatically calculated by the device [20,21]. The parameters included average retinal nerve fiber layer (RNFL) thickness, inferior RNFL thickness, superior RNFL thickness, temporal RNFL thickness, nasal RNFL thickness, average cup/disc ratio (CDR), vertical cup/disc ratio (VCDR), rim area, and disc area.

### 2.3. Glaucoma Diagnosis

Three glaucoma experts (referred to as Expert 1, Expert 2, and Expert 3), each from different institutions and with at least 20 years of clinical experience specializing in glaucoma, independently and comprehensively evaluated the medical records, including medical history and ophthalmic examinations. All participants were categorized into three groups: glaucoma, glaucoma suspects, and non-glaucoma, according to the glaucoma diagnostic criteria based on the classifications from the International Society for Geographical and Epidemiological Ophthalmology (ISGEO), the Collaborative Initial Glaucoma Treatment Study (CGIS), the Advanced Glaucoma Intervention Study (AGIS), and other large-scale glaucoma cohort studies [22,23,24,25,26].

Glaucomatous optic neuropathy (GON) was determined based on optic disc photographs, defined by the following criteria: (1) a vertical cup-to-disc ratio greater than 0.7 for glaucoma or 0.65 or higher for glaucoma suspects; (2) vertical cup-to-disc ratio asymmetry greater than 0.2 for glaucoma or 0.2 or less for glaucoma suspects; (3) a neuroretinal rim width less than 0.1 for glaucoma or less than or equal to 0.1 for glaucoma suspects; and (4) the presence of localized or diffuse RNFL defects on fundus photography.

Glaucomatous VF (GVF) defects were defined as occurring in two or more repeatable and corresponding VF examinations when at least two of the following three conditions were met: (1) the Glaucoma Hemifield Test (GHT) results were outside normal limits; (2) the pattern standard deviation had a *p*-value of less than 0.05; and (3) three or more contiguous points were depressed with a probability *p*-value of less than 5%, including at least one point with a probability *p*-value of less than 1% on the pattern deviation plot.

Glaucoma was defined by the following criteria: (1) the presence of both GON and GVF defects and (2) the presence of typical GON, even if satisfactory VF testing could not be completed, provided there was no alternative explanation for GON and GVF.

Participants were considered glaucoma suspects if they met the GON criteria but did not have definite corresponding GVF defects or if they met the GVF criteria without meeting the GON criteria.

A diagnosis of non-glaucoma was made if neither of the above criteria were met.

The exclusion criteria were as follows: (1) a history of intraocular surgery, except for cataract surgery; (2) penetrating ocular injury, severe blunt ocular trauma, or an abnormal anterior chamber angle; (3) inflammatory eye disease requiring steroid-containing eye drops for more than two weeks; (4) diabetic retinopathy, retinal vein occlusion, or other retinal or optic nerve diseases causing VF defects; (5) evidence of intracranial mass lesions, bleeding, or tumors on a CT examination; (6) severe systemic conditions that impede examination; and (7) inability to obtain informed consent.

All participants were divided into three groups: (1) Group A: where the three glaucoma subspecialists had different diagnoses; (2) Group B: where two glaucoma subspecialists agreed, but the third disagreed; (3) Group C: where all three glaucoma subspecialists were in agreement.

### 2.4. Statistical Analysis

Data were statistically analyzed using SPSS version 22.0 and R version 4.2.1. Normally distributed data were presented as mean ± standard deviation (SD), while non-normally distributed data were presented as median ± interquartile range. Categorical data were presented as number (%). Intergroup comparisons were performed using *t*-tests or the Kruskal–Wallis test. If statistical differences were found among multiple samples, post-hoc pairwise comparisons were conducted, and *p*-values were Bonferroni corrected. The χ^2^ test or Fisher’s exact test was used for categorical variables. In this study, a significant *p*-value was set at < 0.05 (two-tailed). Interobserver agreement between two glaucoma subspecialists was assessed using Cohen’s kappa analysis, while agreement among the three glaucoma subspecialists was assessed using Fleiss’ kappa analysis. Kappa values were interpreted as follows: 0.00 or less indicates poor agreement, 0.01 to 0.20 indicates slight agreement, 0.21 to 0.40 indicates fair agreement, 0.41 to 0.60 indicates moderate agreement, 0.61 to 0.80 indicates substantial agreement, and 0.81 to 1.00 indicates almost-perfect agreement [19].

## 3. Results

Initially, 498 eyes from 285 subjects were recruited from the WGPS. However, 34 eyes were excluded due to unreliable or missing ophthalmic examination results. Consequently, the study comprised 464 eyes from 275 participants, with a median age of 73.0 ± 17.0 years; 144 (52.4%) participants were male, and 131 (47.6%) were female.

The median IOP of the enrolled eyes was 14.42 ± 4.50 mmHg. The mean deviation (MD), pattern standard deviation (PSD), and VF index (VFI) were −4.07 ± 5.51 dB, 3.97 ± 5.26 dB, and 92.75 ± 14.00%, respectively. Mild VF loss (MD < −6.0 dB) was present in 67.89% of eyes (Table 1).

The three glaucoma subspecialists reached a consensus on the diagnosis for 30.17% of the eyes (140). In 59.05% of the eyes (274), two of the three subspecialists agreed, while in 10.78% of the eyes (50), the three subspecialists had completely different diagnoses.

Statistically significant differences were found in intergroup comparisons of IOP, all optic disc parameters (including average RNFL thickness, rim area, average CDR, vertical CDR, cup volume, and RNFL thickness in four quadrants), and all VF parameters (VFI, MD, PSD, and GHT). These results suggest that the degree of VF defects, RNFL thickness, and CDR influenced the experts’ clinical decisions. Agreement among clinicians was more likely when diagnosing eyes with larger average and vertical CDRs, more severe VF defects, and thicker average RNFL thickness, particularly in the temporal and inferior quadrants. High myopia, defined as AL greater than 26 mm or spherical equivalent (SE) less than −6.00 D, also impacted interobserver agreement.

Among the 464 eyes, the rate of glaucoma diagnosis varied between 28.7% and 69.2%, the rate of suspected glaucoma ranged from 22.8% to 34.1%, and the rate of non-glaucoma diagnoses ranged from 6.0–37.3% (Table 2).

Interobserver agreement was highest between Expert 1 and Expert 3, with a kappa value of 0.282 and 67.24% consistency in diagnoses. In contrast, the agreement between Expert 3 and Expert 2 was lowest, with a kappa value of 0.133 and only 39.8% consistency (Table 3 and Table 4). The Fleiss’ kappa for interobserver agreement among all three experts was 0.149.

## 4. Discussion

Previous glaucoma studies have often regarded the clinical diagnoses made by experienced glaucoma specialists as the gold standard. The European Glaucoma Prevention Study Group [27] recommended assessing variability in glaucoma diagnosis by evaluating both intra-observer and interobserver agreement. Given that previous studies have shown moderate to good intra-observer agreement, this study primarily focuses on interobserver agreement. The study recruited participants who met the inclusion criteria from the WGPS cohort study. The interobserver agreement among the three glaucoma experts was notably poor, with a Fleiss’ kappa value of 0.149. The kappa values between the two experts ranged from 0.133 to 0.282. The lowest agreement was observed between Expert 3 and Expert 2, with inconsistent diagnoses in 60.13% of eyes, while the highest agreement was between Expert 1 and Expert 3, with inconsistencies in 32.76% of eyes.

Previous studies have mainly showed poor to moderate interobserver agreement in detecting specific glaucomatous changes with only fundus color photographs or OCT or VF results. In a study by Abrams et al. [16], only moderate interobserver agreement (Fleiss’ kappa value = 0.46) was observed in interpreting fundus photographs among ophthalmologists from the same institution, even with unified glaucoma diagnostic criteria. The agreement on key glaucomatous optic nerve head characteristics—such as enlarged CDR, disc rim narrowing, RNFL loss, and disc hemorrhage—varied from poor to moderate, with kappa values of 0.499. 0.367, 0.188, and 0.89, respectively. Blumberg’s study [18] found poor interobserver agreement for VF and fundus photograph assessments, with kappa values of 0.13 and 0.16, respectively, while agreement for OCT was moderate, with a kappa value of 0.40. There are a few studies exploring interobserver agreement among physicians that included fundus photographs, VF, and OCT results, as far as we know. In Hood’s study [17], which was similar to our design using a self-designed one-page report containing ophthalmic examinations mentioned above, reported moderate interobserver agreement with the kappa value of 0.50 and agreement was achieved in 60% of cases. But Hood’s study had a smaller sample size, including only 50 eyes and kept all information, except VF, fundus photography, and OCT results, confidential, which simplified information processing. Therefore, our study might provide a more accurate reflection of the clinical diagnosis process in real-world settings compared to previous study.

Disagreements can stem from both observer- and subject-related factors. Despite providing standardized diagnostic criteria, our study showed substantial variability among the three experienced glaucoma experts from different institutions. Notably, Expert 2 had a much lower glaucoma diagnosis rate (28.7%) due to a stringent diagnostic threshold. Individual experiences and backgrounds can influence how clinicians interpret and evaluate examination results. Glaucoma subspecialists generally demonstrate higher interobserver agreement compared to non-glaucoma clinicians. Studies by Abrams and Varma [16,28] highlighted that ophthalmologists (kappa value = 0.68) and glaucoma experts (kappa value = 0.65) significantly outperformed optometrists and ophthalmology residents (both with a kappa value = 0.56) in evaluating the CDR. The interobserver agreement for evaluating GON was 0.40 for optometrists, 0.50 for ophthalmology residents, and 0.47 to 0.51 for glaucoma experts. Similarly, Lin et al. found that the glaucoma subspecialists had higher interobserver agreement (kappa value = 0.43–0.60) compared to general ophthalmologists (kappa value = 0.35–0.43) in VF interpretation [29]. Additionally, Breuseg et al. [30] demonstrated that training could improve interobserver agreement and accuracy among non-glaucoma specialist ophthalmologists. In clinical practice, general ophthalmologists often struggle to distinguish glaucoma from physiological cupping, with an inter-examiner agreement of 0.30. However, diagnostic accuracy and inter-rater agreement improve significantly with the addition of VF testing or OCT [14].

Subject-related factors, such as the diverse clinical manifestations of glaucoma, may contribute to the observed diagnostic differences. The degree of VF defects, RNFL abnormalities, and CDR significantly impact diagnostic consistency among glaucoma specialists. Agreement is more readily achieved in cases with relatively severe damage. In addition to the complexity of the disease itself, the methods used for examination can also affect diagnostic results. For instance, Medeiros et al. [31] found that OCT diagnostic performance was significantly influenced by disease severity and optic disc size. The use of deep learning technology may improve clinicians’ ability to evaluate glaucoma more effectively [32]. Furthermore, since glaucoma is a progressive condition, some cases initially classified as suspects or non-glaucoma may develop into glaucoma over time. Therefore, establishing long-term follow-up for eyes with inconsistent diagnoses is advisable.

Statistical significance was also observed in the high myopia group (χ^2^ = 6.257, *p* = 0.043). Population-based studies indicate that the risk of glaucoma increases with the degree of myopia. However, fundus changes associated with high myopia can complicate the diagnosis of glaucoma. There is ongoing debate about whether changes in the optic disc and RNFL due to high myopia should be classified as myopia-related alterations or as indicators of glaucoma. Expert 2 supported the former viewpoint, while other experts held different opinions.

This study acknowledges several limitations regarding its design. It focused solely on interobserver agreement in glaucoma diagnosis without delving into specific glaucomatous manifestations, such as CDR evaluation, RNFL loss, and VF defects. Additionally, the interobserver consistency index did not correspond with specificity and sensitivity indices. The combined use of OCT and VF tests did not enhance sensitivity or specificity compared to using color retinography alone. However, since specificity and sensitivity analyses were based on glaucoma specialists’ diagnoses as the gold standard, this study could not perform such an analysis.

Moreover, we admitted we may underestimate consistency in glaucoma diagnosis, since: (1) The enrolled eyes in our study predominantly had mild VF damage, with 62.6% of cases showing an MD between 0 and −6 dB. The higher proportion of early glaucoma cases could contribute to increased interobserver inconsistencies. (2) Most healthy eyes were excluded from our study since participants were initially screened by two ophthalmology residents. Only eyes with suspicious glaucomatous features were included in the WPGS, which may have heightened the diagnostic challenge. (3) Our study used a more detailed classification system, categorizing eyes into glaucoma, glaucoma suspect, and non-glaucoma. In contrast, many previous studies employed a simpler classification of glaucoma versus non-glaucoma, which might have influenced the interobserver agreement.

## 5. Conclusions

Despite the use of standardized diagnostic criteria for glaucoma, notable variations in interobserver consistency remain among glaucoma subspecialists. To enhance agreement among clinicians, it is advisable to implement training focused on the unified diagnostic criteria. Furthermore, for cases with inconsistent diagnoses, long-term follow-up is crucial to accurately confirm or rule out glaucoma.

## Figures and Tables

**Table 1:** Demographic and Ocular Characteristics of the 464 Eyes Analyzed in the Study.

Characteristic	Group A (*n* = 50)	Group B (*n* = 274)	Group C (*n* = 140)	Total (*n* = 464)	*p*-Value			
						A vs. B	A vs. C	B vs. C
VA, LogMar	0.17 ± 0.21	0.10 ± 0.24	0.12 ± 0.18	0.12 ± 0.22	0.876	-	-	-
IOP, mmHg	14.50 ± 3.71	14.00 ± 4.50	15.25 ± 4.00	14.42 ± 4.50	**0.010**	1.000	0.837	0.08
SE, D	0.06 ± 3.33	0.13 ± 4.25	−0.13 ± 3.97	0.12 ± 4.00	0.552	-	-	-
SE ≥ −6	48 (11.71)	245 (59.76)	117 (28.54)	410 (88.36)	**0.043**	0.110	**0.028**	0.116
SE < −6	2 (3.70)	29 (53.70)	23 (42.59)	54 (11.64)				
AL, mm	23.39 ± 1.73	23.89 ± 2.10	23.91 ± 2.04	23.89 ± 1.99	0.215	-	-	-
AL ≤ 26	47 (12.18)	228 (59.07)	111 (28.76)	386 (83.19)	0.055	**0.033**	**0.026**	0.347
AL > 26	3 (3.85)	46 (58.97)	29 (37.18)	78 (16.81)				
Average RNFL, μm	85.25 ± 14.25	81.00 ± 16.00	68.50 ± 18.00	78.50 ± 19.00	**<0.001**	0.675	**<0.001**	**<0.001**
Rim area, mm^2^	1.09 ± 0.26	1.07 ± 0.39	0.84 ± 0.32	1.01 ± 0.38	**<0.001**	1.00	**<0.001**	**<0.001**
Disc area, mm^2^	2.18 ± 0.70	2.19 ± 0.70	2.09 ± 0.63	2.15 ± 0.67	0.194	-	**-**	**-**
Average CDR	0.70 ± 0.12	0.71 ± 0.14	0.77 ± 0.11	0.72 ± 0.14	**<0.001**	1.00	**<0.001**	**<0.001**
Vertical CDR	0.67 ± 0.12	0.68 ± 0.15	0.78 ± 0.10	0.71 ± 0.17	**<0.001**	1.00	**<0.001**	**<0.001**
Cup volume, mm^3^	0.32 ± 0.33	0.34 ± 0.35	0.47 ± 0.31	0.36 ± 0.35	**<0.001**	1.00	**0.027**	**<0.001**
Superior RNFL, μm	100.25 ± 33.75	99.50 ± 28.00	90.50 ± 34.50	97.25 ± 31.00	**<0.001**	0.876	0.339	**<0.001**
Temporal RNFL, μm	65.25 ± 14.50	63.00 ± 12.63	57.25 ± 17.88	61.50 ± 17.00	**<0.001**	1.00	**<0.001**	**<0.001**
Nasal RNFL, μm	61.50 ± 16.00	63.50 ± 15.00	59.50 ± 14.63	61.50 ± 14.00	**0.026**	1.00	0.599	**0.021**
Inferior RNFL, μm	112.00 ± 24.63	99.00 ± 37.00	67.75 ± 24.63	92.00 ± 43.38	**<0.001**	0.10	**<0.001**	**<0.001**
VFI, %	96.50 ± 6.75	94.50 ± 8.50	81.50 ± 25.38	92.75 ± 14.00	**<0.001**	0.159	**<0.001**	**<0.001**
MD, dB	−2.63 ± 3.96	−3.61 ± 3.56	−7.75 ± 9.17	−4.07 ± 5.51	**<0.001**	0.270	**<0.001**	**<0.001**
PSD, dB	2.76 ± 3.12	3.21 ± 3.49	7.825 ± 7.55	3.97 ± 5.26	**<0.001**	0.223	**<0.001**	**<0.001**
GHT, number (%)								
Outside normal limits	35 (70.00)	203 (74.09)	128 (91.43)	366 (78.88)	**<0.001**	0.273	**<0.001**	**<0.001**
Borderline	4 (8.00)	33 (12.04)	4 (2.86)	41 (8.84)				
Within normal limits	11 (22.00)	38 (13.87)	8 (5.71)	57 (12.28)				

VA: visual acuity; LogMar: logarithm of the minimum angle of resolution; IOP: intraocular pressure; SE: spherical equivalent; AL: axial length; RNFL: retinal nerve fiber layer thickness; CDR: cup-to-disc ratio; VFI: visual field index; MD: mean deviation; PSD: pattern standard deviation; GHT: Glaucoma Hemifield Test.

**Table 2:** Diagnostic Rates for Glaucoma, Suspected Glaucoma, and Non-Glaucoma by Three Experts.

	Glaucoma	Glaucoma Suspect	Non-Glaucoma
Expert 1	315 (67.9%)	121 (26.1%)	28 (6.0%)
Expert 2	133 (28.7%)	158 (34.1%)	173 (37.3%)
Expert 3	321 (69.2%)	106 (22.8%)	37 (8.0%)

**Table 3:** Kappa Values for Interobserver Agreement in Glaucoma Diagnosis.

	Expert 1	Expert 2	Expert 3
Expert 1	-	0.175	0.282
Expert 2		-	0.133
Expert 3			-

**Table 4:** Proportion of Eyes with Consistent Diagnoses Between Pairs of Experts.

	Expert 1	Expert 2	Expert 3
Expert 1	-	197 (42.46%)	312 (67.24%)
Expert 2		-	185 (39.87%)
Expert 3			-

## Data Availability

The raw data supporting the conclusions of this article will be made available by the authors on request.
